# Association of high-estimated glomerular filtration rate with the severity of ischemic stroke during non-vitamin K antagonist oral anticoagulants therapy: a nationwide cohort study

**DOI:** 10.3389/fneur.2023.1277855

**Published:** 2023-12-01

**Authors:** Min Kyoung Kang, Dongwhane Lee, Mi Sun Oh, Ji-Sung Lee, Han-Yeong Jeong, Jung Hwan Shin, Byung-Woo Yoon, Jong-Moo Park

**Affiliations:** ^1^Department of Neurology, College of Medicine, Ewha Womans University, Seoul, Republic of Korea; ^2^Department of Neurology, Uijeongbu Eulji Medical Center, Gyeonggi, Republic of Korea; ^3^Department of Neurology, Hallym University Sacred Heart Hospital, Anyang, Gyeonggi, Republic of Korea; ^4^Clinical Research Center, Asan Institute for Life Sciences, Seoul, Republic of Korea; ^5^Department of Neurology, Seoul National University Hospital, Seoul, Republic of Korea

**Keywords:** acute ischemic stroke, non-vitamin K antagonist oral anticoagulant, high estimated glomerular filtration rate, nationwide multicenter study, glomerular hyperfiltration, high estimated glomerular filtration rate

## Abstract

**Aim:**

While the relationship between impaired kidney function and non-vitamin K antagonist oral anticoagulants (NOACs) is well established, there is limited research exploring the association between an elevated estimated glomerular filtration rate (eGFR) and the efficacy of NOACs, especially concerning the outcomes of acute ischemic stroke (AIS). This study aimed to examine the association between higher-than-normal eGFR and the severity of AIS during the use of NOACs using a nationwide multicenter stroke registry in Korea.

**Material and methods:**

This study utilized data from the Korean Stroke Registry (KSR) database, examining information from 2,379 patients with AIS, who had atrial fibrillation (AF) and a history of utilizing NOACs prior to hospitalization due to incident stroke occurring between 2016 and 2021. Patients with a history involving two or more types of anticoagulants or one or more forms of antiplatelet agents were excluded. Baseline characteristics, medical history, medication usage, CHADS_2_-VASc score, and the anticoagulation and risk factors in atrial fibrillation (ATRIA) score were evaluated. Renal function was assessed using eGFR levels and calculated with the Cockcroft–Gault equation. The severity of stroke was measured by the National Institutes of Health Stroke Scale as an outcome. For sensitivity analysis, further evaluation was performed using eGFR levels according to the modification of diet in renal disease (MDRD) study equation.

**Results:**

The mean age of subjects was 76.1 ± 8.9 years. The moderate-to-severe stroke severity group exhibited an elevation in creatinine levels. The eGFR of 60 to 89 mL/min/1.73 m^2^ group was associated with a decreased risk of moderate-to-severe stroke severity [hazard ratio (HR)] (0.77, 95% confidence interval (CI) [0.61, 0.98], *p* = 0.031) compared to the eGFR≥90 mL/min/1.73 m^2^ group. An increment of 10 units in eGFR was marginally associated with an increased risk of moderate-to-severe stroke severity (HR: 1.03, 95% CI [1.00, 1.07], *p* = 0.054).

**Conclusion:**

The study revealed that individuals with eGFR ≥ 90 mL/min/1.73 m^2^ had an association linked to an increased risk of moderate-to-severe stroke severity. Our study suggests that patients taking NOACs with higher-than-normal eGFR levels may have an increased severity of AIS.

## Introduction

Renal function has been linked with a spectrum of cardiovascular diseases, including acute ischemic stroke (AIS), atrial fibrillation (AF), myocardial infarction, and heart failure. Chronic kidney disease, characterized predominantly by diminished estimated glomerular filtration rate (eGFR), is strongly associated with the risk of these cardiovascular diseases (1, 2). In addition to low eGFR, abnormally high eGFR has also been linked to various health conditions. While high eGFR is generally considered a normal physiological state, it can also indicate underlying adverse renal conditions, such as the onset of glomerular damage in hypertensive patients or diabetic nephropathy (3). High eGFR is also closely intertwined with hypertension, prediabetes, and obesity, all of which may contribute to cardiovascular events (4). In addition, recent studies have reported an association between high eGFR and the poor outcome of cardiovascular disease, including AIS (5, 6).

Non-vitamin K antagonist oral anticoagulants (NOACs) stand as the first-choice drugs for major conditions entailing a risk of thromboembolism in AF (7). It is widely known that patients with impaired kidney function have an increased risk of bleeding and necessitate dose adjustments while undergoing treatment with NOACs (8). However, there is controversy regarding the relationship between elevated eGFR and the efficacy of NOACs (9–12).

Limited studies have examined the relationship between high eGFR and the efficacy of NOACs, especially concerning outcomes of AIS. This study aimed to investigate the association between higher-than-normal eGFR and the risk of increased severity of AIS during the use of NOACs in a nationwide multicenter registry study in Korea.

## Materials and methods

### Data source

The data for this study were derived from the Korean Stroke Registry (KSR) database. Established in 1999, the KSR has been operating as a prospective, multicenter, collaborative hospital-based stroke registry (13). To ensure the consistency and accuracy of data, dedicated auditors engaged in monthly reviews, address queries, and made corrections from researchers (14–16).

The KSR database comprehensively encompasses information on demographic characteristics, medical and medication history, stroke subtype, side of stroke involvement, lesion of stroke involvement, stroke outcomes, and treatment details of patients with AIS. The database entails measuring physical parameters such as height, weight, blood pressure, conducting laboratory tests, and surveying lifestyle habits (14–16).

### Study population

Between January 2016 and December 2020, a total of 85,499 patients diagnosed with AIS (within 7 days of onset) were enrolled from 39 hospitals across Korea. From this registry, we selected the patients with AF and the documented history of administration of NOACs prior to the index stroke. We excluded the patients with a history involving the use of two or more types of anticoagulants, one or more forms of antiplatelet agents, and those with incomplete data at least one variable required to calculate eGFR. Ultimately, 2,379 patients were included in the analysis.

### Definitions and variables

Baseline characteristics were comprehensively assessed, including age, sex, body weight, body mass index, and comorbidities such as hypertension, diabetes mellitus, hyperlipidemia, congestive heart failure, peripheral artery disease, coronary heart disease, transient ischemia attack (TIA), stroke, and active cancer. The assessment was based on the diagnosis and medication record at admission for the index stroke. The side of the stroke involvement was divided into left, right, or multiple, and with regard to the lesion, it was divided into anterior circulation, posterior circulation, or multiple circulation. Smoking status was dichotomized as non-smoker/former smoker or current smoker. The medication history within 7 days preceding the index stroke was collected, including types of NOACs, anti-hypertensive drugs, antidiabetic drugs, and anti-hyperlipidemia agents. The dosages of NOACs or other agents were not recorded as it was determined based on the judgment of the medical professionals. Stroke subtype, the modified Rankin Scale (mRS) prior to admission, initial the National Institutes of Health Stroke Scale (NIHSS) score, and mRS at discharge were recorded in the KSR database (17, 18).

Laboratory assessments in the KSR database were based on the initial blood test results conducted in the emergency department for patients presenting with stroke symptoms, encompassing BUN (blood urea nitrogen), creatinine, leukocyte count, hemoglobin, hematocrit, platelet count, prothrombin time, CRP (C-reactive protein), LDL (low-density lipoprotein), HbA1c (glycated hemoglobin), and D-dimer. The estimated glomerular filtration rate (eGFR) was calculated using the serum creatinine level and Cockcroft–Gault equations (19).

The Cockcroft–Gault equation estimating creatinine clearance (Ccr) was calculated as eGFR (mL/min/1.73 m^2^):


$$\begin{array}{l} \text{ eGFR }=\;\left(140-\text{age}\right)\;\times \text{ weight }\left(\text{kg}\right)\\ \times \;{\left[72\;\times \text{ serum creatinine }\left(\text{mg}/\text{dL}\right)\right]}^{-1}\;\left(\text{if male}\right) \end{array}$$

$$\begin{array}{l} \text{ eGFR }=\;\left(140-\text{age}\right)\;\times \text{ weight }\left(\text{kg}\right)\\ \times \;{\left[72\;\times \text{ serum creatinine }\left(\text{mg}/\text{dL}\right)\right]}^{-1}\;\times \;0.85\;\left(\text{if female}\right). \end{array}$$

We calculated the CHADS_2_-VASc score and the anticoagulation and risk factors in atrial fibrillation (ATRIA) bleeding score in order to assess the risk of occurrence of stroke and the bleeding risk in each patient (20, 21). For sensitivity analysis, further evaluation was performed using eGFR levels according to the modification of diet in renal disease (MDRD) study equation (22).

The modification of diet in renal disease (MDRD) study equation was calculated as eGFR (mL/min/1.73 m^2^):


$$\begin{array}{l} \text{eGFR }=\;186.3\;\times \text{ serum creatinine }{\left(\text{mg}/\text{dL}\right)}^{-1.154}\\ \;\times \;{\text{age}}^{-0.203}\;\left(\text{if male}\right) \end{array}$$

$$\begin{array}{l} \text{eGFR }=\;186.3\;\times \text{ serum creatinine }{\left(\text{mg}/\text{dL}\right)}^{-1.154}\\ \;\times \;{\text{age}}^{-0.203}\;\times \;0.742\;\left(\text{if female}\right). \end{array}$$

The primary outcome was the initial stroke severity at admission, measured by the NIHSS score, with a range from 0 to 42 (23). A mild initial stroke severity was defined as an NIHSS score of 0 to 7 (24). To perform sensitivity analysis, we conducted additional assessments using the mRS scale at discharge. We categorized moderate-to-severe stroke severity as mRS scores ≥3, and death was defined as an mRS score of 6 upon discharge (25).

### Statistical analysis

The study presented data in two formats: mean ± standard deviation or as number and percentage. The chi-square test, Fisher's exact test, Cochran–Mantel–Haenszel shift test, Student's *t*-test, and Wilcoxon rank sum test were employed to compare baseline characteristics between the two severity groups. The relationship between eGFR and initial stroke severity was examined by categorizing patients into ranges of eGFR (< 30, 30–59, 60–89, and ≥90 mL/min/1.73 m^2^), with the range of >90 mL/min/1.73 m^2^ serving as the reference group (26).

Multivariable logistic regression models were applied to investigate the factors that affected moderate-to-severe initial stroke severity. These analyses were adjusted for potential confounding variables including age, sex, hypertension, diabetes mellitus, hyperlipidemia, congestive heart failure, coronary heart disease, peripheral arterial disease, previous TIA or stroke, active cancer, current smoking status, history of taking NOACs, stroke subtype, laboratory data, the CHADS_2_-VASC score, and ATRIA score to identify the independent contributing power of kidney function for the use of NOACs. Potential determinants based on the results of univariate analysis (*P* < 0.1) and variables that were already known to be related to NOAC therapy were selected for multivariate analysis. Odds ratios (OR) with 95% CI were reported.

Subgroup analyses were conducted based on a stroke subtype or previous stroke history. For sensitivity analysis, we conducted additional assessments which included evaluating eGFR levels using the MDRD study equation and using mRS scores ≥3 or death at discharge as additional indicators of stroke severity.

Statistical analyses were conducted using SAS software (version 9.2, SAS Institute, Cary, NC, USA), with a *p* < 0.05 considered statistically significant.

### Ethical approval statement

The Institutional Review Board of Uijeongbu Eulji Medical Center approved this study and provided a consent waiver (Institutional Review Board approval number: 2022–07–004), as the KSR permitted restricted access to anonymized data for research purposes.

## Results

The average age of the patients with AIS was 76.1 ± 8.9 years, and male subjects accounted for 50.0% of the cohort. The prevalence of hypertension, diabetes mellitus, and hyperlipidemia was 85.1, 35.4, and 57.4%, respectively. In this study, we observed that the moderate-to-severe stroke severity group tended to be older, have lower body weight and BMI, and a lesser use of anti-hypertensive drugs and anti-diabetes drugs. Additionally, we found a lower prevalence of male, hyperlipidemia, and a previous TIA history among individuals in the moderate-to-severe stroke severity group. The side of the lesion did not show statistically significant differences, but when considering the location of the lesion, the moderate-to-severe stroke severity of stroke was higher in cases of anterior circulation infarction. Elevated BUN, decreased creatinine levels, higher leukocyte counts, CRP levels, and D-dimer levels were observed as well as lower hemoglobin, prothrombin time, and HbA1c levels compared to the mild severity group. Notably, there was no significant difference in eGFR levels upon comparison by means between the two groups, while lower creatinine levels were evident in the moderate-to-severe stroke severity group. The CHADS_2_-VASC score and ATRIA score, which determined stroke occurrence or complication risk in patients with atrial fibrillation, were higher in the moderate-to-severe stroke severity group (Table 1).

**Table 1:** Baseline characteristics of study population.

	**NOAC users**		***P-value***
	**Mild stroke severity (NIHSS**<**8)**	**Moderate to severe stroke severity (NIHSS** ≥**8)**	
Number of patients	1,438 (60.4)	941 (39.6)	
**Demographics**			
Age, years	75.2 ± 9.0	77.5 ± 8.5	< 0.001
Male	798 (55.5)	392 (41.7)	< 0.001
Body weight, kg	62.7 ± 11.7	59.3 ± 12.2	< 0.001
Body mass index, kg/m^2^	23.8 ± 3.6	23.0 ± 3.9	< 0.001
**Comorbidities**			
Hypertension	1,229 (85.5)	795 (84.5)	0.511
Diabetes mellitus	525 (36.5)	318 (33.8)	0.176
Hyperlipidemia	860 (59.8)	506 (53.8)	0.004
Congestive heart failure	59 (4.1)	61 (6.5)	0.010
Peripheral artery disease	14 (1.0)	9 (1.0)	0.967
Coronary heart disease	250 (17.4)	172 (18.3)	0.577
Previous TIA history	56 (3.9)	18 (1.9)	0.007
Previous stroke history	699 (48.6)	463 (49.2)	0.777
Active cancer history	62 (4.3)	33 (3.5)	0.327
Current smokers	133 (9.2)	66 (7.0)	0.054
**Side of stroke involvement**	1,214 (84.4)	881(93.6)	0.279
Left	534 (44.0)	401 (45.5)	
Right	508 (41.8)	340 (38.6)	
Multiple	172 (14.2)	140 (15.9)	
**Lesion of stroke involvement**	1,214 (84.4)	881(93.6)	< 0.001
Anterior circulation	715 (58.9)	690 (78.3)	
Posterior circulation	379 (31.2)	88 (10.0)	
Multiple	120 (9.9)	103 (11.7)	
**Stroke subtype**			< 0.001
Cardioembolism	887 (61.7)	769 (81.7)	
Other etiology	551 (38.3)	172 (18.3)	
**Previous medication**			
Anti-hypertensive drug	1,076 (74.8)	655 (69.6)	0.005
Anti-diabetic drug	430 (29.9)	230 (24.4)	0.004
Anti-hyperlipidemia agent	707 (49.2)	438 (46.5)	0.211
**Neurological status**			< 0.001
Premorbid mRS			
0	943 (65.6)	529 (56.2)	
1	226 (15.7)	127 (13.5)	
2	122 (8.5)	82 (8.7)	
3	95 (6.6)	90 (9.6)	
4	48 (3.3)	68 (7.2)	
5	4 (0.3)	45 (4.8)	
Initial NIHSS, median (IQR)	2 (1–4)	15 (11–19)	< 0.001
Discharge mRS			< 0.001
0	282 (19.6)	26 (2.8)	
1	393 (27.3)	61 (6.5)	
2	274 (19.1)	87 (9.2)	
3	244 (17.0)	124 (13.2)	
4	167 (11.6)	231 (24.5)	
5	66 (4.6)	318 (33.8)	
6	11 (0.8)	94 (10.0)	
**Kidney function marker**			
BUN, mg/dL	18.7 ± 8.0	20.1 ± 10.3	0.001
Creatinine, mg/dL	1.01 ± 0.55	0.96 ± 0.40	0.013
eGFR			
Cockroft-Gauld, mL/min/1.73 m^2^	73.1 ± 26.2	73.9 ± 27.5	0.474
MDRD, mL/min/1.73 m^2^	77.8 ± 27.9	78.6 ± 29.3	0.474
Laboratory marker			
Leukocyte count, x10^3^/μL	7.5 ± 2.5	8.4 ± 3.1	< 0.001
Hemoglobin, g/dL	13.3 ± 2.2	12.8 ± 2.2	< 0.001
Hematocrit, %	39.9 ± 9.7	39.5 ± 19.3	0.543
Platelet count, x10^3^/μL	205.6 ± 70.1	207.4 ± 74.1	0.546
Prothrombin Time, sec/INR	1.32 ± 1.62	1.21 ± 0.37	0.014
C-reactive protein, mg/dL	0.3 (0.1–1.2)	0.7 (0.2–3.9)	< 0.001
Low-density lipoprotein, mg/dL	87.8 ± 31.9	85.6 ± 29.4	0.072
HbA1c, %	6.2 ± 1.1	6.0 ± 1.0	< 0.001
D-dimer, μg/mL	0.5 (0.3–1.2)	1.2 (0.6–3.0)	< 0.001
CHADS_2_-VASC score	4.32 ± 1.52	4.58 ± 1.49	< 0.001
ATRIA score	3.03 ± 2.06	3.26 ± 2.11	0.008

Data are expressed as the mean ± standard deviation, or number (percentage). P-value by Chi-square test, Fisher's exact test, CMH shift test, Student's t-test and Wilcoxon rank sum test. NOAC, non-vitamin K antagonist oral anticoagulants; NIHSS, National Institutes of Health Stroke Scale; TIA, transient ischemia attack; mRS, modified Rankin Scale; IQR, interquartile range; BUN, blood urea nitrogen; eGFR, estimated glomerular filtration rate; MDRD, Modification of Diet in Renal Disease; INR, International normalized ratio; Atria, Anticoagulation and Risk Factors in Atrial Fibrillation.

**Figure 1:**
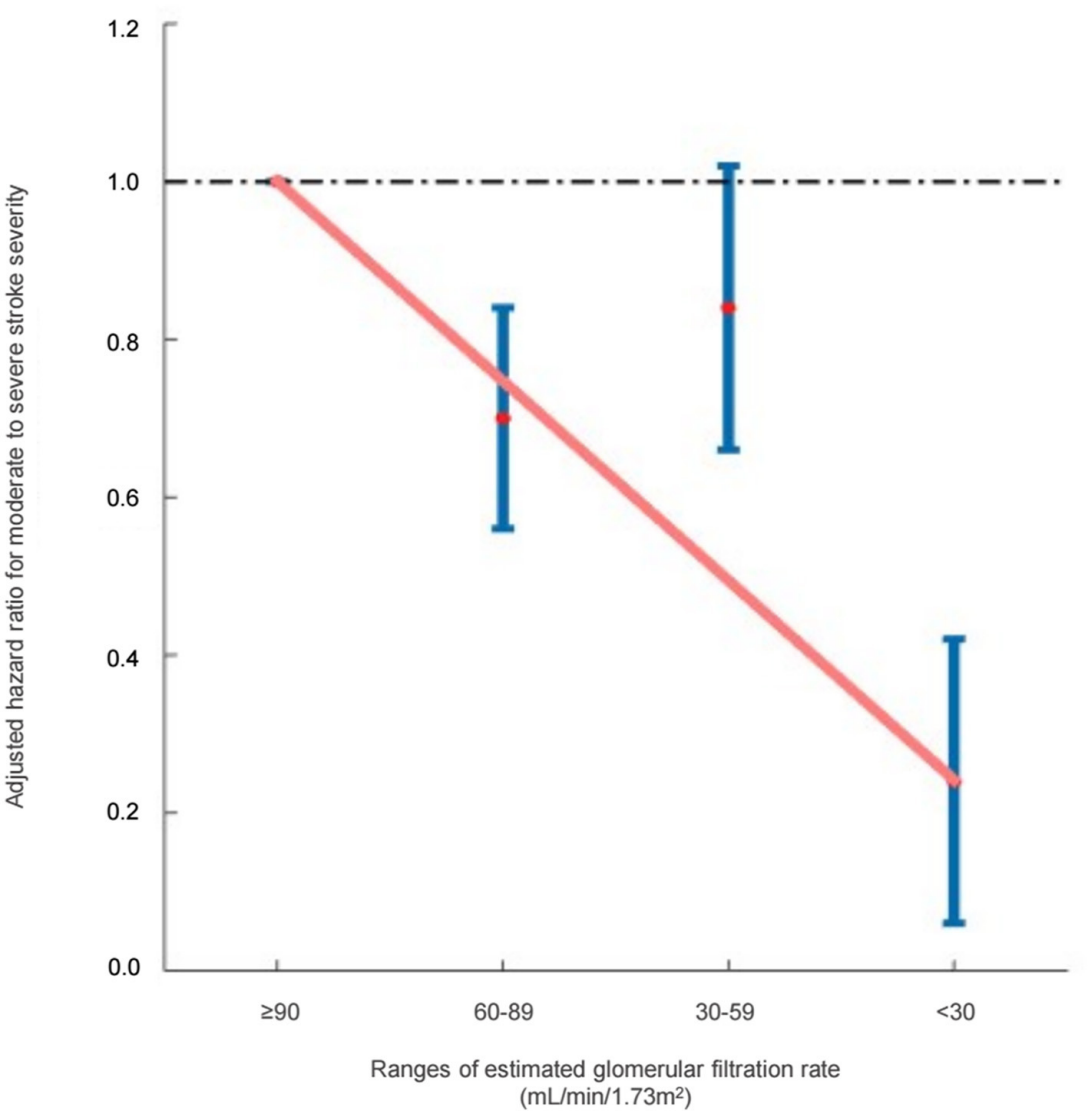
Hazard ratios for moderate-to-severe stroke severity based on the ranges of estimated glomerular filtration rate (Cockcroft–Gault equation).

With respect to renal function, 21.0% of the patients with mild stroke severity and 25.1% of the patients with moderate-to-severe stroke severity fell into the eGFR≥90 mL/min/1.73 m^2^ group (reference), exhibiting higher-than-normal eGFR levels. After being adjusted for comorbidities and laboratory results, an eGFR of 60–89 mL/min/1.73 m^2^ (HR: 0.77, 95% CI [0.61, 0.98], *p* = 0.031) and eGFR of < 30 mL/min/1.73 m^2^ (HR: 0.42, 95% CI [0.22, 0.77], *p* = 0.005) were associated with a decreased risk of moderate-to-severe stroke severity, whereas an eGFR of 30–59 mL/min/1.73 m^2^ (HR: 0.91, 95% CI [0.69, 1.18], *p* = 0.466) was not associated with a decreased risk of moderate-to-severe stroke severity (Table 2). The hazard ratio plot demonstrated a bimodal decline in the hazard ratio for stroke severity as GFR decreased (Figure 1).

**Table 2:** Comparison of kidney function according to stroke severity.

	**Mild stroke severity (NIHSS < 8)**	**Moderate to severe stroke severity (NIHSS ≥8)**	***P* ^†^**	**Unadjusted OR (95% CI)**	**P**	**Adjusted OR (95% CI)**	***P* ^‡^**
Number of patients	1,438	941					
eGFR, mL/min/1.73 m^2^ (Cockroft-Gauld)			< 0.001				
≥90	302 (21.0)	236 (25.1)		1(Ref)		1(Ref)	
60–89	713 (49.6)	388 (41.2)		0.70 (0.56–0.86)	< 0.001	0.77 (0.61–0.98)	0.031
30–59	379 (26.4)	295 (31.3)		1.00 (0.79–1.25)	0.973	0.91 (0.69–1.18)	0.466
< 30	44 (3.1)	22 (2.3)		0.64 (0.37–1.10)	0.105	0.42 (0.22–0.77)	0.005

^†^P-value by Chi-square test.^‡^P-value by logistic regression [adjusted for age, sex, history of hypertension, diabetes mellitus, hyperlipidemia, congestive heart failure, peripheral artery disease, coronary heart disease, previous stroke history, current smoke, side of stroke involvement, lesion of stroke involvement and laboratory results (WBC, Hb, HCT, PLT, INR, LDL)]. NIHSS, National Institutes of Health Stroke Scale; OR, odds ratio; CI, confidence interval; eGFR estimated glomerular filtration rate; MDRD, Modification of Diet in Renal Disease.

In the multivariate analysis, a 10 unit increase in eGFR (Cockroft-Gault) was marginally associated with an increased risk of moderate-to-severe stroke severity (HR: 1.03, 95% CI [1.00, 1.07], *p* = 0.054) (Table 3). In addition, higher age, female, and absence of hyperlipidemia were significantly associated with stroke severity. When it comes to the stroke lesion, the side of the stroke did not have a significant correlation. Posterior circulation infarction was negatively associated moderate-to-severe strokes compared to anterior circulation infarction. When considering stroke subtypes, cases of small-vessel occlusion and strokes with an undetermined etiology exhibited a negative association with moderate-to-severe strokes in comparison to those attributed to large artery atherosclerosis.

**Table 3:** Predictors for moderate to severe stroke severity in patients with non-vitamin K antagonist oral anticoagulants.

**Variations**	**Univariate model**		**Multivariate model**	
	**OR (95% CI)**	***P*** **-value**	**OR (95% CI)**	***P-*** **value**
**Kidney function (Cockroft-Gauld)**				
eGFR (mL/min/1.73 m^2^, per 10 unit increase)	1.01 (0.98–1.04)	0.472	1.03 (1.00–1.07)	0.054
Age (per 10 unit increase)	1.35 (1.23–1.49)	< 0.001	1.25 (1.11–1.41)	< 0.001
Male	0.57 (0.48–0.68)	< 0.001	0.64 (0.52–0.78)	< 0.001
Hypertension	0.93 (0.74–1.16)	0.509	0.92 (0.70–1.20)	0.539
Diabetes mellitus	0.89 (0.75–1.05)	0.176	0.99 (0.81–1.21)	0.915
Hyperlipidemia	0.78 (0.66–0.92)	0.004	0.80 (0.65–0.97)	0.022
Congestive heart failure	1.62 (1.12–2.34)	0.010	1.46 (0.94–2.24)	0.089
Coronary heart disease	1.06 (0.86–1.32)	0.576	1.05 (0.82–1.34)	0.711
Peripheral artery disease	0.98 (0.42–2.28)	0.967	0.98 (0.38–2.54)	0.974
Previous TIA/stroke history	1.02 (0.87–1.21)	0.777	1.15 (0.95–1.40)	0.155
Active cancer	0.81 (0.52–1.24)	0.328	0.94 (0.59–1.52)	0.810
Current smoker	0.74 (0.54–1.01)	0.055	1.07 (0.75–1.55)	0.698
**History of NOAC**				
Apixaban	0.79 (0.66–0.95)	0.012	0.53 (0.21–1.34)	0.179
Dabigatran	1.16 (0.91–1.46)	0.231	0.76 (0.30–1.93)	0.563
Edoxaban	0.89 (0.74–1.07)	0.230	0.56 (0.22–1.41)	0.217
Ribaroxaban	1.29 (1.07–1.55)	0.007	0.73 (0.29–1.84)	0.507
**Side of stroke involvement**				
Left	1(Ref)		1(Ref)	
Right	0.89 (0.74–1.08)	0.240	0.92 (0.75–1.13)	0.443
Multiple	1.08 (0.84–1.40)	0.540	1.33 (0.98–1.81)	0.063
**Lesion of stroke involvement**				
Anterior circulation	1(Ref)		1(Ref)	
Posterior circulation	0.24 (0.19–0.31)	< 0.001	0.25 (0.19–0.33)	< 0.001
Multiple	0.89 (0.67–1.18)	0.418	0.74 (0.54–1.03)	0.073
**Stroke subtype**				
Large artery atherosclerosis	1(Ref)		1(Ref)	
Small vessel occlusion	0.01 (0.00–0.24)	0.003	0.02 (0.00–0.41)	0.010
Cardioembolism	0.93 (0.60–1.45)	0.752	1.04 (0.65–1.68)	0.868
Other etiology	1.31 (0.33–5.23)	0.700	1.30 (0.28–5.96)	0.735
Undetermined	0.38 (0.24–0.62)	< 0.001	0.49 (0.29–0.82)	0.007

OR, odds ratio; CI, confidence interval; eGFR, estimated glomerular filtration rate; TIA, transient ischemia attack; NOAC, non-vitamin K antagonist oral anticoagulants.

**Figure 2:**
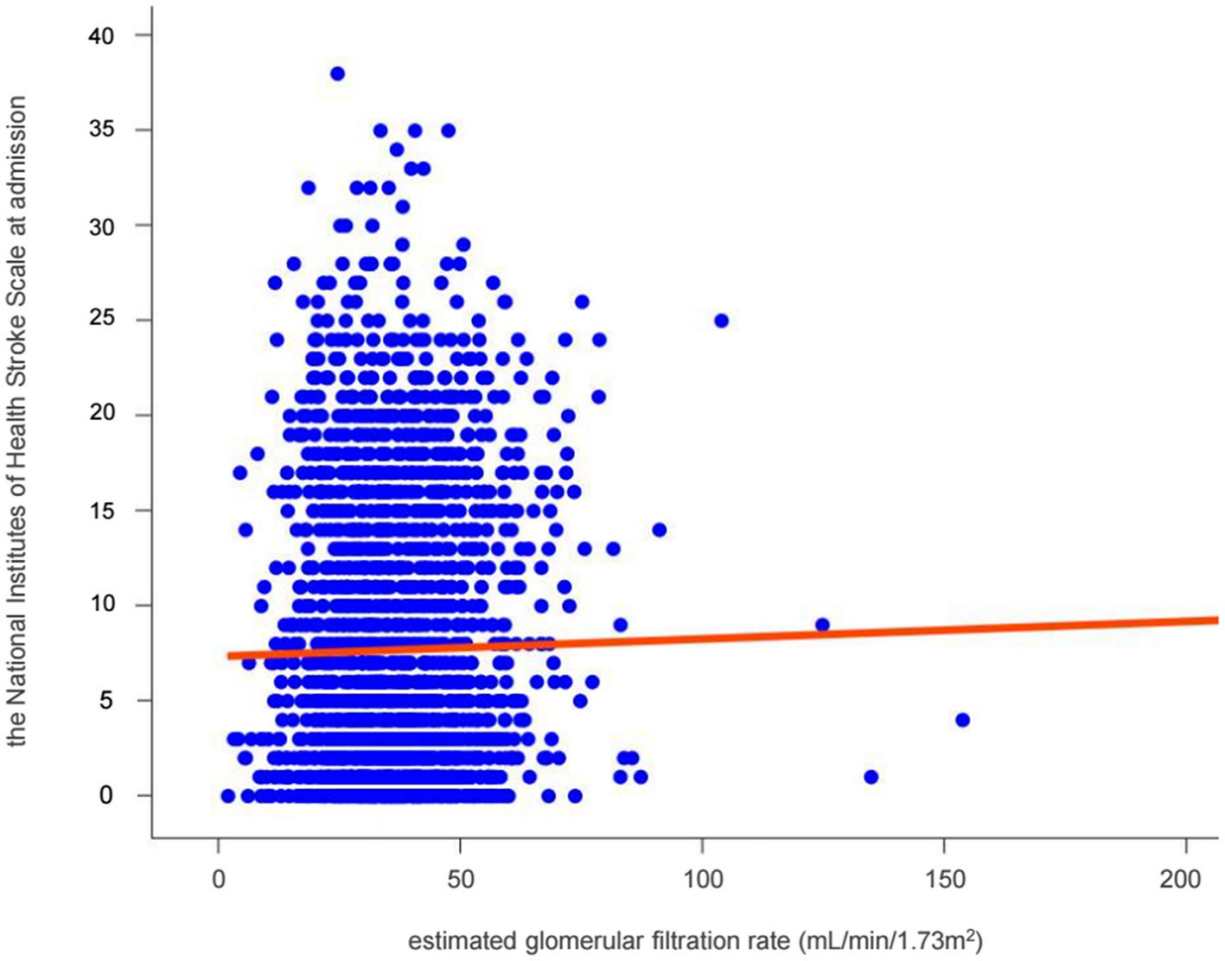
Relationship between renal function and stroke severity in patients with non-vitamin K antagonist oral anticoagulants. The solid red line represents the multivariate-adjusted linear regressions.

The scatter plot demonstrated an incline in the NIHSS score which represents stroke severity, as GFR increased (Figure 2).

Subgroup analysis revealed no significant interactions between eGFR levels and stroke severity based on the stroke subtype. However, there was significant interaction based on previous TIA or stroke history (Table 4). In the presence of previous TIA or stroke history, a 10 unit increase in eGFR was significantly associated with an increased risk of moderate-to-severe stroke severity (HR: 1.07, 95% CI [1.01, 1.13], *p* = 0.013).

**Table 4:** Subgroup analysis of predictors for moderate to severe stroke severity in patients with non-vitamin K antagonist oral anticoagulants.

**Variations**	**Univariate model**		**Multivariate model**		**Univariate model**		**Multivariate model**	
	**OR (95% CI)**	***P-*** **value**	**OR (95% CI)**	***P-*** **value**	**OR (95% CI)**	***P-*** **value**	**OR (95% CI)**	***P-*** **value**
**Stroke subtype**	**Cardioembolism**				**Other than cardioembolism**			
**Kidney function (Cockroft-Gauld)**								
eGFR (mL/min/1.73 m^2^, per 10 unit increase)	1.02(0.98–1.06)	0.408	1.03 (1.00–1.08)	0.117	0.99 (0.92–1.06)	0.714	1.06 (0.98–1.14)	0.150
Age (per 10 unit increase)	1.29 (1.15–1.44)	< 0.001	1.20 (1.05–1.37)	0.007	1.69 (1.33–2.15)	< 0.001	1.67 (1.24–2.25)	< 0.001
Male	0.60 (0.50–0.73)	< 0.001	0.64 (0.51–0.80)	< 0.001	0.47 (0.32–0.68)	< 0.001	0.54 (0.35–0.84)	0.006
Hypertension	0.92 (0.70–1.20)	0.536	0.93 (0.69–1.26)	0.632	0.95 (0.54–1.65)	0.852	0.87 (0.46–1.65)	0.672
Diabetes mellitus	0.85 (0.69–1.04)	0.122	0.87 (0.70–1.09)	0.240	1.17 (0.81–1.69)	0.407	1.55 (1.01–2.36)	0.044
Dyslipidemia	0.84 (0.69–1.03)	0.089	0.88 (0.71–1.10)	0.264	0.61 (0.43–0.89)	0.009	0.49 (0.32–0.75)	0.001
Congestive heart failure	1.42 (0.94–2.14)	0.096	1.61 (0.99–2.61)	0.054	1.61 (0.65–3.96)	0.300	1.18 (0.39–3.55)	0.763
Coronary heart disease	1.04 (0.81–1.34)	0.756	1.00 (0.75–1.32)	0.982	1.25 (0.78–2.01)	0.348	1.29 (0.75–2.23)	0.363
Peripheral artery disease	0.66 (0.19–2.25)	0.505	0.46 (0.13–1.65)	0.233	1.86 (0.58–5.95)	0.296	2.60 (0.63–10.73)	0.185
Previous TIA/stroke history	1.01 (0.83–1.22)	0.944	1.14 (0.92–1.42)	0.236	1.09 (0.76–1.57)	0.647	1.21 (0.80–1.84)	0.366
Active cancer	0.67 (0.40–1.13)	0.135	0.86 (0.49–1.52)	0.607	1.45 (0.66–3.22)	0.357	1.18 (0.49–2.88)	0.708
Current smoker	0.85 (0.59–1.22)	0.378	1.22 (0.80–1.85)	0.360	0.48 (0.24–0.97)	0.042	0.79 (0.34–1.79)	0.566
**History of NOAC**								
Apixaban	0.83 (0.67–1.03)	0.086	0.55 (0.21–1.46)	0.227	0.83 (0.57–1.21)	0.335	0.43 (0.02–7.51)	0.565
Dabigatran	1.16 (0.88–1.52)	0.290	0.78 (0.29–2.12)	0.629	1.08 (0.62–1.88)	0.790	0.67 (0.04–12.10)	0.785
Edoxaban	0.89 (0.72–1.11)	0.306	0.58 (0.22–1.54)	0.273	0.79 (0.52–1.21)	0.284	0.47 (0.03–8.28)	0.609
Ribaroxaban	1.22 (0.99–1.52)	0.067	0.74 (0.28–1.99)	0.556	1.48 (0.98–2.23)	0.060	0.75 (0.04–13.06)	0.844
**Side of stroke involvement**								
Left	1(Ref)		1(Ref)		1(Ref)		1(Ref)	
Right	0.89 (0.71–1.10)	0.282	0.89 (0.70–1.12)	0.313	1.02 (0.68–1.52)	0.943	0.97 (0.62–1.50)	0.874
Multiple	0.82 (0.61–1.09)	0.176	1.17 (0.83–1.63)	0.373	2.76 (1.53–4.96)	< 0.001	2.78 (1.35–5.70)	0.005
**Lesion of stroke involvement**								
Anterior circulation	1(Ref)		1(Ref)		1(Ref)		1(Ref)	
Posterior circulation	0.22 (0.16–0.29)	< 0.001	0.21 (0.15–0.28)	< 0.001	0.39 (0.24–0.64)	< 0.001	0.37 (0.22–0.62)	< 0.001
Multiple	0.69 (0.50–0.94)	0.021	0.64 (0.45–0.92)	0.017	2.13 (1.13–4.01)	0.019	1.43 (0.66–3.13)	0.367
**Previous TIA/stroke history**	**Yes**				**No**			
**Kidney function (Cockroft-Gauld)**								
eGFR (mL/min/1.73 m^2^, per 10 unit increase)	1.038 (0.994–1.083)	0.088	1.07 (1.01–1.13)	0.013	0.98 (0.94–1.03)	0.421	1.00 (0.95–1.05)	0.941
Age (per 10 unit increase)	1.38 (1.20–1.59)	< 0.001	1.23 (1.03–1.46)	0.020	1.33 (1.16–1.53)	< 0.001	1.24 (1.05–1.46)	0.011
Male	0.59 (0.46–0.74)	< 0.001	0.64 (0.48–0.85)	0.002	0.56 (0.44–0.70)	< 0.001	0.62 (0.47–0.81)	< 0.001
Hypertension	0.93 (0.68–1.28)	0.661	1.02 (0.71–1.47)	0.919	0.92 (0.66–1.29)	0.632	0.78 (0.53–1.15)	0.216
Diabetes mellitus	0.99 (0.78–1.27)	0.945	1.00 (0.76–1.32)	0.982	0.80 (0.62–1.02)	0.066	0.86 (0.65–1.13)	0.267
Dyslipidemia	0.63 (0.49–0.80)	< 0.001	0.61 (0.46–0.81)	< 0.001	0.91 (0.72–1.15)	0.431	0.95 (0.73–1.23)	0.680
Congestive heart failure	1.61 (0.90–2.88)	0.112	2.07 (1.02–4.20)	0.043	1.64 (1.02–2.63)	0.041	1.35 (0.78–2.36)	0.285
Coronary heart disease	1.16 (0.84–1.60)	0.366	1.22 (0.85–1.76)	0.284	0.99 (0.74–1.33)	0.969	0.94 (0.68–1.31)	0.720
Peripheral artery disease	1.14 (0.39–3.29)	0.815	1.45 (0.43–4.93)	0.551	0.77 (0.19–3.10)	0.715	0.43 (0.10–1.78)	0.242
Active cancer	1.09 (0.59–2.02)	0.782	1.20 (0.61–2.37)	0.591	0.61 (0.33–1.13)	0.119	0.75 (0.39–1.45)	0.393
Current smoker	0.67 (0.43–1.04)	0.071	0.79 (0.47–1.32)	0.362	0.82 (0.53–1.26)	0.361	1.27 (0.76–2.12)	0.355
**History of NOAC**								
Apixaban	0.82 (0.64–1.05)	0.116	0.70 (0.19–2.55)	0.589	0.77 (0.60–0.99)	0.044	0.28 (0.07–1.18)	0.082
Dabigatran	1.02 (0.74–1.39)	0.910	0.90 (0.24–3.32)	0.876	1.36 (0.95–1.95)	0.094	0.51 (0.12–2.21)	0.366
Edoxaban	0.95 (0.72–1.25)	0.720	0.85 (0.23–3.10)	0.801	0.85 (0.66–1.09)	0.202	0.28 (0.07–1.20)	0.088
Ribaroxaban	1.31 (1.00–1.72)	0.048	1.00 (0.27–3.66)	0.998	1.27 (0.99–1.64)	0.063	0.39 (0.09–1.67)	0.207
**Side of stroke involvement**								
Left	1(Ref)		1(Ref)		1(Ref)		1(Ref)	
Right	0.93 (0.71–1.21)	0.578	0.95 (0.71–1.27)	0.741	0.86 (0.66–1.12)	0.275	0.86 (0.65–1.14)	0.296
Multiple	1.15 (0.79–1.67)	0.461	1.61 (1.04–2.50)	0.033	1.03 (0.72–1.47)	0.883	1.33 (0.87–2.03)	0.183
**Lesion of stroke involvement**								
Anterior circulation	1(Ref)		1(Ref)		1(Ref)		1(Ref)	
Posterior circulation	0.24 (0.17–0.35)	< 0.001	0.22 (0.15–0.32)	< 0.001	0.24 (0.17–0.34)	< 0.001	0.23 (0.16–0.33)	< 0.001
Multiple	0.80 (0.54–1.19)	0.270	0.67 (0.43–1.06)	0.085	1.00 (0.66–1.51)	0.989	0.85 (0.53–1.36)	0.491

OR, odds ratio; CI, confidence interval; eGFR, estimated glomerular filtration rate; MDRD, Modification of Diet in Renal Disease; TIA, transient ischemia attack; NOAC, non-vitamin K antagonist oral anticoagulants.

Sensitivity analysis consistently showed that an eGFR of 60–89 mL/min/1.73 m^2^ (HR: 0.75, 95% CI [0.61, 0.93], *p* = 0.007) and eGFR of <30 mL/min/1.73 m^2^ (HR: 0.37, 95% CI [0.20, 0.71], *p* = 0.003) were associated with a decreased risk of moderate-to-severe stroke severity, and the marginal association of eGFR levels and severity of stroke was also observed (HR: 1.03, 95% CI [1.00, 1.06], *p* = 0.050) even when eGFR levels were assessed using the MDRD method (Supplementary Tables 1, 2). In the sensitivity analysis, where an mRS score ≥3 was used as an indicator of neurological severity, it was found that eGFR of 60–89 mL/min/1.73 m^2^ (HR: 0.69, 95% CI [0.55, 0.87], *p* = 0.001) and eGFR of 30–59 mL/min/1.73 m^2^ (HR: 0.68, 95% CI [0.53, 0.88], *p* = 0.003) were associated with a decreased risk of moderate-to-severe stroke severity, while there was no association with in-hospital mortality (Supplementary Tables 3–1, 3–2). In the multivariate analysis, a 10 unit increase in eGFR was not associated with an increased risk of moderate-to-severe stroke severity defined as the mRS score of 3 or above (HR: 1.04, 95% CI [1.00, 1.07], *p* = 0.057) as well as for the patients with in-hospital mortality (Supplementary Tables 4–1, 4–2).

## Discussion

Our study revealed that individuals with eGFR ≥ 90 mL/min/1.73 m^2^ demonstrated an association linked to an increased risk of moderate-or-severe stroke severity, which remains significant in individuals presenting with previous TIA or stroke history.

Numerous studies have investigated the relationship between eGFR and stroke. A multicenter-based prospective cohort-based study which was involving ~500,000 individuals, revealed that the hazard ratio for death at 1 year or severe stroke severity as defined by Barthel index <75, increased in decreased eGFR groups, with a proportional relationship between the extent of renal function impairment and a higher occurrence of death or severe sequelae (27). Contrarily, previous studies have reported “U” or “J”-shaped relationships between eGFR and stroke severity or mortality, suggesting that both low and high eGFR levels are associated with an increased mortality risk. A multicentre population-based stroke registry-based study conducted in Japan, involving ~1,400,000 individuals, revealed that the hazard ratio for all in-hospital death and at-discharge death/disability increased in eGFR < 45 mL/min/1.73 m^2^ and eGFR ≥ 90 mL/min/1.73 m^2^ groups (28).

When compared to normal eGFR levels, a relatively high eGFR was linked to an increased risk of cardiovascular diseases and mortality. In a prior study of the chronic kidney disease epidemiology collaboration dataset, participants with a high eGFR, defined as 95th percentiles of the age- and sex-specific eGFR quintile (HR 1.5, 95% CI [1.2–2.1]) exhibited a significantly heightened risk of cardiovascular events compared to those with normal eGFR (29). In a prospective cohort study comprising 16,958 participants without clinically evident vascular disease, an eGFR >90 mL/min/1.73 m^2^ was associated with a high risk for coronary heart disease and non-vascular mortality compared to the normal eGFR group (5). Furthermore, a retrospective study of roughly 43,500 individuals from a general population health screening cohort with a mean observation period of 12.4 years identified a correlation between high eGFR and increased risk of cardiovascular-associated mortality after adjustment for the age-, sex-, muscle mass-, and history of diabetes and/or hypertension (30). In summary, high eGFR levels may be associated with poor outcomes of cardiovascular events, including stroke, compared to normal eGFR levels.

Generally, although a high eGFR is frequently deemed favorable due to its indication of good kidney function, it can serve as a marker for underlying health conditions such as hypertension, diabetes, and obesity. As these conditions worsen, the risk of cardiovascular events and mortality increases. In our results, the mean eGFR did not differ between the mild and moderate/severe stroke severity groups before adjustment (Table 1). However, the association between high eGFR and increased risk of stroke was significant after adjusting cardiovascular risk factors. Furthermore, even when the criterion for moderate-to-severe stroke severity was changed to a discharge mRS score of 3 or above, a similar pattern was observed although a direct association with mortality was not evident (Supplementary Tables 3–1, 3–2). Since eGFR is the result of combining the effects of complex factors such as hypertension, diabetes, and BMI as well as renal hyperfiltration, the effect of renal function on stroke severity must be judged after controlling for confounding factors.

In our results, high eGFR exhibited a higher risk of moderate-to-severe stroke, as shown in the eGFR ≥ 90 mL /min/1.73 m^2^ group. High eGFR may be associated with dysfunction of the renin-angiotensin system, low-grade vascular or systemic inflammation, endothelial dysfunction, and increased arterial stiffness. These factors represent the primary mechanisms contributing to the development of cardiovascular/cerebrovascular disease and increased mortality risk (29, 31, 32). Another possible mechanism is that hyperfiltration may lead to enhanced removals, causing suboptimal plasma concentration of NOACs, first doubted by the ENGAGE AF-TIMI 48 study, which reported a higher ischemic stroke rate for edoxaban in patients with creatinine clearance > 95 mL/min (HR 1.45, 95% CI [0.90–2.35]). Similar findings were derived from subanalyses of apixaban and rivaroxaban trials (33, 34). However, pharmacokinetic studies have reported no significant alterations in hyperfiltrated patients with NOACs (35). Therefore, it seems more plausible to consider that poor outcomes of cardiovascular events in hyperfiltration patients could stem from the adverse effects of an early stage of chronic kidney disease as important and independent cardiovascular risk factors, rather than sole alteration of pharmacokinetics. In our subgroup analysis results, the fact that the effect of renal hyperfiltration was more evident in the group with a history of TIA or stroke, regardless of stroke side, lesion, or etiology including cardioembolism, could support the hypothesis that systemic involvement of renal hyperfiltration was the major component, rather than the change of concentration of NOACs induced by hyperfiltration. Other results of Mendelian analysis served as supplementary evidence. A previous Mendelian analysis study, conducted for myocardial infarction which was another type of cardiovascular disease, highlighted renal hyperfiltration as a causal risk factor for poor outcomes (36). Further investigation is necessary to substantiate the hypothesis.

Our research has limitations that should be acknowledged. First, there may have been a potential for ethnic selection bias, which could limit the applicability of our results to other populations. Additional research on diverse races and ethnicities is warranted (16). Second, we only assessed cross-sectional eGFR and did not perform cystatin C measurements, which were not included in the KSR dataset. Third, the dosages of NOACs or other agents were determined based on the medical professionals' discretion. Lastly, a retrospective nature of this study might impede the establishment of a causal relationship.

## Conclusion

In summary, this study revealed that individuals with eGFR ≥ 90 mL/min/1.73 m^2^ demonstrated an association linked to an increased risk of moderate-to-severe stroke severity. Our study suggests that patients taking NOACs with higher-than-normal eGFR levels may have an increased severity of AIS.

## Data availability statement

The raw data supporting the conclusions of this article will be made available by the authors, without undue reservation.

## Ethics statement

The studies involving humans were approved by the Institutional Review Board of Uijeongbu Eulji Medical Center approval number: 2022–07–004. The studies were conducted in accordance with the local legislation and institutional requirements. Written informed consent for participation was not required from the participants or the participants' legal guardians/next of kin in accordance with the national legislation and institutional requirements.

## Author contributions

MK: Conceptualization, Investigation, Writing—original draft, Writing—review & editing. DL: Conceptualization, Supervision, Writing—original draft. MO: Conceptualization, Data curation, Formal analysis, Project administration, Writing— original draft. J-SL: Conceptualization, Data curation, Methodology, Validation, Writing—original draft. H-YJ: Data curation, Writing—original draft. JS: Methodology, Software, Validation, Writing—original draft. B-WY: Methodology, Project administration, Writing—original draft. J-MP: Conceptualization, Writing—original draft, Writing—review & editing.
